# Role of T Regulatory Cells and Myeloid-Derived Suppressor Cells in COVID-19

**DOI:** 10.1155/2022/5545319

**Published:** 2022-04-19

**Authors:** Alhasan Alsalman, Mohammad A. Al-Mterin, Eyad Elkord

**Affiliations:** ^1^Natural and Medical Sciences Research Center, University of Nizwa, Nizwa, Oman; ^2^Biomedical Research Center, School of Science, Engineering and Environment, University of Salford, Manchester, UK

## Abstract

Coronavirus disease 2019 (COVID-19) has been raised as a pandemic disease since December 2019. Immunosuppressive cells including T regulatory cells (Tregs) and myeloid-derived suppressor cells (MDSCs) are key players in immunological tolerance and immunoregulation; however, they contribute to the pathogenesis of different diseases including infections. Tregs have been shown to impair the protective role of CD8^+^ T lymphocytes against viral infections. In COVID-19 patients, most studies reported reduction, while few other studies found elevation in Treg levels. Moreover, Tregs have a dual role, depending on the different stages of COVID-19 disease. At early stages of COVID-19, Tregs have a critical role in decreasing antiviral immune responses, and consequently reducing the viral clearance. On the other side, during late stages, Tregs reduce inflammation-induced organ damage. Therefore, inhibition of Tregs in early stages and their expansion in late stages have potentials to improve clinical outcomes. In viral infections, MDSC levels are highly increased, and they have the potential to suppress T cell proliferation and reduce viral clearance. Some subsets of MDSCs are expanded in the blood of COVID-19 patients; however, there is a controversy whether this expansion has pathogenic or protective effects in COVID-19 patients. In conclusion, further studies are required to investigate the role and function of immunosuppressive cells and their potentials as prognostic biomarkers and therapeutic targets in COVID-19 patients.

## 1. Introduction

Coronavirus disease 2019 (COVID-19) is caused by SARS-CoV-2. In December 2019, the first cases were reported in China, and the virus quickly spread to other countries around the world [1, 2]. In less than three years, SARS-CoV-2 has infected hundreds of millions. There are a wide variety of clinical symptoms, ranging from asymptomatic to severe symptoms, with acute respiratory distress syndrome (ARDS) and multiorgan dysfunction in fewer than 10% of patients [3, 4]. Several factors increase the risk of COVID-19 disease, including aging, high blood pressure, cardiovascular disease, diabetes, and obesity [5]. The virus causes early immunological suppression through unknown mechanisms. Lymphocyte subsets, particularly CD4^+^ and CD8^+^ T cells, were altered in COVID-19 patients, and lymphopenia has been reported as the primary symptom in most cases of COVID-19 patients [6]. It has been shown that lymphopenia was worsened with the progression of disease to respiratory distress syndrome [7].

T regulatory cells (Tregs) play critical roles in immunological tolerance, but they also contribute to the pathogenesis of different diseases, including cancer, autoimmune diseases, transplantation, and infections. Myeloid-derived suppressor cells (MDSCs) are immature myeloid cells that strongly suppress the immune system by inhibiting different immune cells, including T cells, natural killer cells (NK), and dendritic cells [8–11]. COVID-19 pathogenesis and severity could be linked to dysregulation of immunosuppressive cells to SARS-CoV-2 [8]. In this review, we present the available data describing the role of Tregs and MDSCs in viral infections and COVID-19 patients.

## 2. T Regulatory Cells

Tregs have important roles in the modulation of immune responses by maintaining self-tolerance and immunological homeostasis. They contribute to the regulation of immune responses to many diseases [12]. They can suppress various immune cells, including CD4^+^ and CD8^+^ T cells, monocytes, dendritic cells, B cells, and NK cells, to reduce unwanted immune responses in different immune diseases such as allergy, autoimmunity, and transplant rejection [13–15]. Generally, Tregs are classified into two types based on their origin: thymus-derived Tregs (tTreg) and peripherally induced Tregs (pTreg) [16, 17]. tTregs originate in the thymus and migrate to the periphery to control peripheral immunological tolerance [18]. pTregs are induced in peripheral tissues, and they differentiate by contact with nonself-antigens in the presence of transforming growth factor *β* and IL-2 [19, 20]. Tregs express different molecules essential for their function such as CD25, cytotoxic T lymphocyte associated antigen-4 (CTLA-4), and forkhead box P3 (FoxP3) [12]. The FoxP3 is a transcriptional factor, which is essential for Treg development and function. Immunosuppressive activities of FoxP3+superscript Tregs could be determined by the level of FoxP3 expression [21]. Tregs were classified into three subgroups based on FoxP3 and CD45RA expression; these include activated Tregs (CD45RA^−^FoxP3^high^), resting Tregs (CD45RA^+^FoxP3^low^), and non-Tregs (CD45RA^−^FoxP3^low^). Activated Tregs strongly inhibit immune responses, compared with resting Tregs. However, non-Tregs secrete different effector cytokines such as interferon (IFN)-*γ*, interleukin (IL)-2, and IL-17 but without any inhibitory function [22, 23].

### 2.1. Role of Tregs in Viral Infection

Herein, we briefly discuss some studies investigated Tregs in viral infections in human. It has been demonstrated that the presence of Tregs impaired the protective function of CD8^+^ T cells against viral infection [24, 25]. There has been a variety of explanations for Treg suppressive ability in viral infections, including a decrease in the quantity of the protective T cell responses, a reduction in the antiviral cytokine secretion by effector cells, and preventing the migration of protective T cells to the infected region [26]. According to Raiden et al., Tregs were reduced in the peripheral blood of infected infants with severe respiratory syncytial virus (RSV), this could be explained by elevated levels of Tregs in lung and lymph nodes and increased apoptosis [27]. Another study reported that Tregs, TGF-*β*, and IL-10 were decreased in infants with RSV bronchiolitis infection, compared with healthy infants [28]. Additionally, Qin et al., reported that bronchial epithelial cells of infected humans with RSV inhibited the differentiation of Treg subsets and induced the differentiation of Th2 and Th17 cells [29]. Some studies reported that CD4^+^CD25^+^ T cells were increased in chronic hepatitis C virus (HCV) disease, compared with recovered or normal patients [30–32]. These data indicated that the inhibition of virus-specific CD8^+^ T cells was increased in patients with chronic HCV disease; this could be associated with elevated levels of Tregs in HCV patients [32]. Also, some researchers indicated that Tregs were increased in liver and peripheral blood of HCV patients [33]. Other studies reported that Tregs and Th17 were increased in infected patients with chronic hepatitis B virus (HBV) [34–36]. Additionally, elevated Tregs in peripheral blood were associated with HBV replication in chronic disease, and it was less common in the early stages of acute HBV infection [37]. Another study showed that frequency of Tregs was significantly increased in chronic active HBV and asymptomatic HBV carriers, in comparison to resolved and controlled patients [38, 39]. However, Prendergast et al. found that the count of Tregs was decreased in human immunodeficiency virus (HIV) patients [40]. Interestingly, the count of Tregs was decreased in the blood of HIV patients, but the proportion of Tregs was increased in chronic infection, which could be associated with HIV progression [41]. Milman et al. reported that the count of Tregs was significantly high in herpes simplex virus type 2 (HSV-2), compared with control biopsy from unaffected skin [42].

### 2.2. Role of Tregs in COVID-19

**Figure 1:**
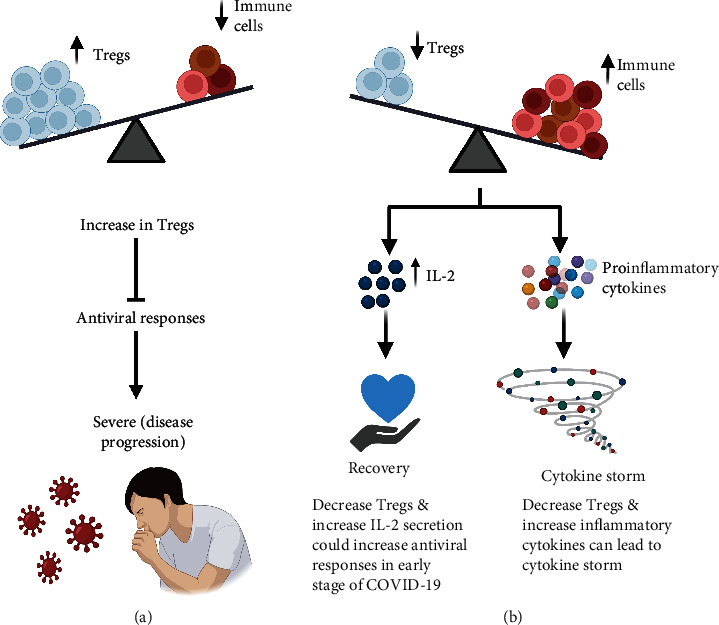
Role of Tregs in COVID-19 patients: An increase in level of Tregs could play a negative role in COVID-19 patients by inhibiting antiviral T cell responses, resulting in the progression of COVID-19 disease (a). A decrease in level of Tregs could result in cytokine storm or recovery depending on the response of proinflammatory cytokines. Firstly, early increase in IL-2 could be associated with faster viral clearance and early immune response in asymptomatic COVID-19 patients. Secondly, the significant increase in T helper cells such as Th17 could be related to hyperinflammation and progression of COVID-19 disease (b).

There have been different findings for investigating Tregs in COVID-19 patients. Firstly, Tregs and the transcription factor FoxP3 were elevated in severe COVID-19 patients, which was associated with worse outcomes [43]. These data indicate that Tregs could play negative roles in COVID-19 by inhibiting antiviral T cell responses in the severe phases of illness (Figure 1(a)). Moreover, in critical COVID-19 patients, the activity and frequency of Tregs were increased, compared with other respiratory diseases such as influenza and respiratory syncytial virus (RSV) (Table 1) [44]. Interestingly, IL-10-secreting Tregs, a lineage known to possess anti-inflammatory properties in the lung, were elevated in severe COVID-19 patients, compared to mild/moderate diseases [45]. These results indicate that the increase in IL-10-secreting Tregs could contribute to more severe COVID-19 symptoms. In addition, in mechanically ventilated COVID-19 patients, the percentage of Tregs and Th17 cells was highly increased in the lung, compared with blood [46]. Some studies indicated that activated CD4^+^CD25^+^CD127^low^ Tregs were highly increased in moderate and severe COVID-19 patients, compared with healthy controls [47, 48]. De Biasi et al. reported that Tregs and IL-10 were elevated in the blood of COVID-19 patients [49]. A potential explanation for such increase of Tregs in circulation is that SARS-CoV-2 impeded the transit of Tregs from circulation to the respiratory tract, resulting in Treg accumulation in circulation and lung damage due to excessive inflammatory response in the lung [44].

Secondly, Rezaei et al. reported that the total counts of white blood cells, T cells, CD38^+^, and CD3^+^HLA-DR^+^ lymphocytes were significantly elevated in hospitalized COVID-19 patients [50]. Also, they found that CD4^+^/CD8^+^ ratio, B cells, FoxP3^+^ Tregs, and FoxP3 median fluorescence did not show any significant difference between early and late responders of hospitalized COVID-19 patients (Table 1) [50].

Thirdly, Kratzer et al. showed that CD4^+^ and CD8^+^ T effector memory cells, plasma blast, and transitional B cells were elevated in convalescent COVID-19 patients. However, CD25^+^FoxP3^+^ Tregs were significantly decreased in convalescent COVID-19 patients, compared with healthy donors [51]. In line with these findings, Sadeghi et al. found that the count of Tregs and the expression level of FoxP3, TGF-*β*, and IL-10 were decreased in critical COVID-19 patients, compared with healthy controls [52]. In contrast, patients had a significant increase in Th17 cells and associated cytokines IL-17 and IL-23 in COVID-19 patients [52]. These data indicate that the increased and decreased responses of Th17 and Tregs, respectively, could be strongly correlated with hyperinflammation and pathogenesis of the disease (Figure 1(b)). Additionally, Patterson et al. reported that T cells expressing PD-1 and Tregs were highly reduced in COVID-19 patients, compared with healthy controls [53]. A recent study reported that asymptomatic COVID-19 patients have a reduction in Tregs and anti-inflammatory cytokine IL-10 [54]. Also, they found that the early increase in inflammatory cytokine IL-2 was associated with faster viral clearance and early immune responses in asymptomatic COVID-19 patients (Figure 1(b)) [54]. According to Meckiff et al. the cytotoxic follicular helper cells and cytotoxic T helper cells were increased in hospitalized COVID-19 patients [55]. They also reported that Tregs were decreased in hospitalized compared to nonhospitalized patients [55]. These data indicate that immunosuppressive Tregs were impaired in hospitalized COVID-19 patients [55]. Another study reported that T lymphocytes and Tregs were significantly decreased in critical COVID-19 patients with ARDS, compared with severe illness [56]. However, they also found that the percentage of CD45RO^+^CD95^+^ Tregs, among other Treg subsets, was increased in critical COVID-19 patients, compared with severe patients (Table 1) [56]. Additionally, it has been reported that expression level of CD4^+^FoxP3^+^CD25^+^ was significantly decreased in COVID-19 patients, compared with healthy controls [57]. Other studies reported that Tregs were decreased in moderate adult and pediatric patients, and Tregs were more reduced in severe COVID-19 patients [58, 59]. Furthermore, Tregs and activated T cells were decreased in hospitalized COVID-19 patients, compared to healthy controls [8]. Moreover, COVID-19 patients have lower levels of Tregs (CD4^+^CD25^+^CD127^low^), especially in severe cases of the disease [59]. Levels of Tregs were significantly decreased in severe COVID-19 patients, compared with moderate and mild illness [60, 61]. Interestingly, levels of Tregs were increased through the progression from mild to severe patients but then decreased through the progression to critical illness (Table 1) [62]. Moreover, activated CD4^+^ T cells in severe COVID-19 patients expressed higher levels of CD25, while suppressing the expression of FoxP3, resulting in a disrupted FoxP3-mediated mechanism in the lung [63].

In severe COVID-19 patients, lower levels of Tregs could be one of the explanations for the hyperactivated immune system and injured lungs. These reductions in Tregs in COVID-19 patients could be explained by some potential mechanisms. Kalfaoglu et al. reported that IL-2 transcripts were decreased in severe COVID-19 patients, compared with mild illness [63]. Therefore, decreased IL-2 could enhance the apoptosis of Tregs. Moreover, levels of soluble IL-2R (CD25) were increased in severe COVID-19 patients, which could lead to binding of IL-2 with its receptor (IL-2R) and enhance apoptosis of Tregs [59, 61].

### 2.3. Role of Tregs in COVID-19 Elderly Patients

Old age is one of the most important risk factors in COVID-19, and the majority of COVID-19-related deaths are in elderly patients [64]. The severity of COVID-19 in elderly patients could be associated with age-related thymic involutions and consequent T cell changes [65]. In the elderly, increased ratio of thymic Tregs (tTregs) to thymic T conventional cells (tTcon) [66] results in exacerbated age-related accumulation of peripheral Tregs (pTreg) [67–69]. Accumulation of pTregs in the elderly impairs immunological balance and inhibits antiviral immune responses [65]. In COVID-19, early inflammatory response is crucial for host protection. Therefore, older patients are probably not capable of mounting strong antiviral immune responses in the early stages, which leads to increased viral load and damage associated with inflammation. Overall, a weak early inflammatory response is associated with severe symptoms in older age, while a robust early inflammatory response is associated with asymptomatic or mild illness [54].

## 3. Myeloid-Derived Suppressor Cells (MDSCs)

Generally, MDSCs in human are defined as CD33^+^CD11b^+^HLA-DR^low^ cells and are classified into two primary subgroups based on differences in cell morphology and cell-surface markers: granulocytic (polymorphonuclear) CD33^+^CD11b^+^HLA-DR^low^CD15^+^ cells (G-MDSCs) and monocytic CD33^+^CD11b^+^HLA-DR^low^CD14^+^ cells (M-MDSCs) [70–73]. More recently, an additional subgroup has been identified as CD33^+^CD11b^+^ HLA-DR^low^CD14^−^CD15^−^, and they are named immature or early-stage MDSCs (e-MDSCs) [72, 74, 75].

MDSCs present at very low levels in healthy individuals because of the rapid differentiation into mature myeloid cells. However, in the presence of pathological conditions, such as malignancies, infections, bone marrow transplantation, or some autoimmune diseases, MDSC levels are highly increased due to inhibition of their differentiation into mature myeloid cells [76, 77]. Interestingly, when activated in a pathogenic situation, these cells overexpress immune inhibitory factors such as nitric oxide synthase (NOS), arginase 1 (ARG1), and peroxynitrite (ONOO^−^) [76, 78]. Additionally, MDSCs have the ability to increase the number of FoxP3^+^ Tregs [76, 79].

MDSCs have been shown to expand in the peripheral blood of individuals suffering from a variety of malignant and nonmalignant illnesses [80]. Indeed, MDSC levels in cancer patients are considered to have prognostic and predictive value [81]. MDSC subpopulations of monocytic and granulocytic cells have been identified and characterized in different human cancers [82–85]. They inhibit antitumor immune responses [85–87], and as a result, cancer cells continue to evolve [75].

### 3.1. MDSCs in Viral Infections

Levels of MDSCs are elevated in viral diseases, and they could potently suppress T cell proliferation and decrease viral clearance [9, 88, 89]. MDSCs were found to be significantly higher in the blood of chronic hepatitis C (CHC) patients, compared with healthy controls [88]. Interestingly, they found that HCV-RNA levels in plasma were related to the amount of MDSCs in CHC patients [88]. Furthermore, Tacke et al. showed that hepatitis C virus (HCV) enhanced the accumulation of MDSCs, resulting in a decrease in T cell responses [90]. Specifically, Ren et al. observed an expansion in M-MDSCs, but not G-MDSCs, in chronic HCV-infected patients [91]. Garg et al. found that MDSC levels were elevated in activated and nonactivated HIV-infected patients [92]. Moreover, Vollbrecht et al. observed a higher level of G-MDSC in chronic HIV-1 patients, compared with healthy controls. Also, they found a positive relationship between MDSC frequencies and viral load and a negative relationship with CD4^+^ amount in HIV-1 patients [93]. In addition, Pal et al. showed that MDSCs have been linked to T cell dysfunction in infected patients with chronic hepatitis B virus (HBV) [94]. However, Pallett et al. observed that G-MDSCs have a protective mechanism by expressing arginase I to effectively inhibit HBV-specific T cell responses in hepatitis B virus- (HBV-) infected patients [95].

### 3.2. MDSCs in COVID-19

#### 3.2.1. Pathogenic Roles

**Figure 2:**
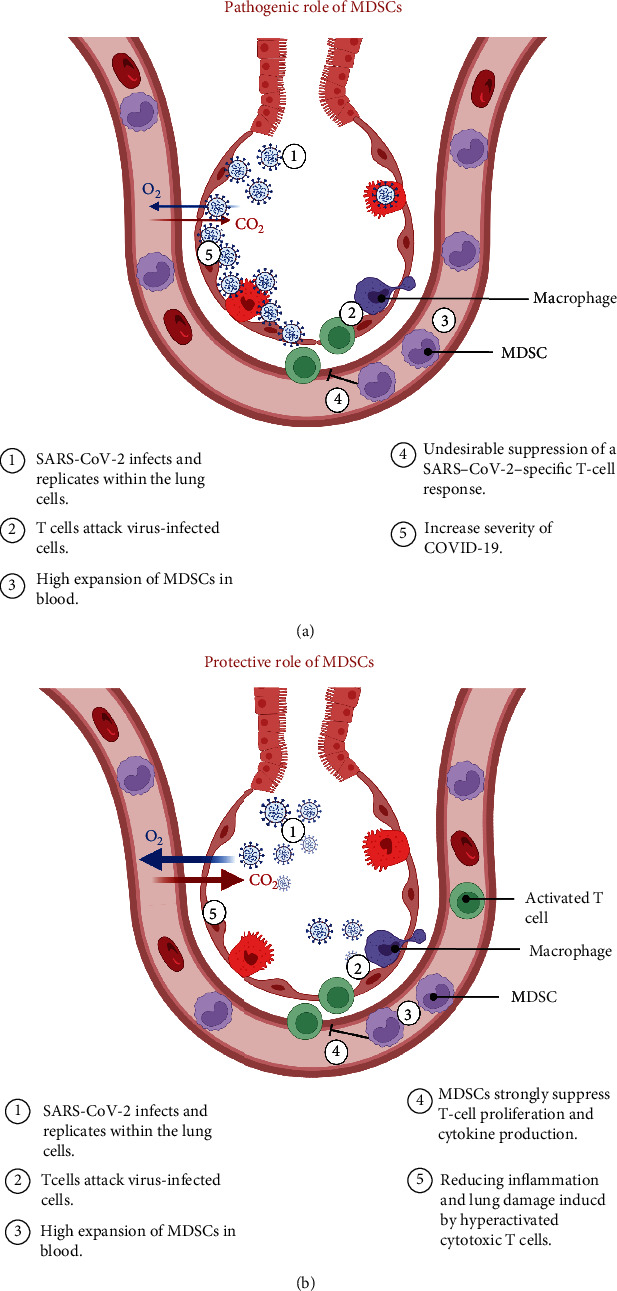
Pathogenic and protective roles of MDSCs in COVID-19 patients: Severe COVID-19 patients had higher levels of MDSCs. This might result in an undesirable suppression of SARS-CoV-2-specific T cell responses, which can contribute to worse outcomes in these patients (a). MDSC expansion may help to limit an overly aggressive and possibly damaging immune responses by decreasing inflammation caused by hyperactivated T cells (b).

In COVID-19 patients, different studies observed that alteration in MDSC levels in the blood has been associated with disease severity (Figure 2(a)) [96, 97]. Furthermore, Reizine et al. found that expansion of MDSCs in response to COVID-19 was shown to be significantly linked to lymphopenia and increased arginase activity [98]. Also, they found that frequency of MDSCs in severe COVID-19 patients was higher in hospitalized patients than patients with moderate COVID-19 [98]. Accordingly, it has been reported that SARS-CoV-2 patient plasma inhibited human leukocyte antigen D related (HLA-DR) expression [99]. Therefore, decreasing levels of HLA-DR on monocytes have been observed in severe COVID-19, considering an increase in M-MDSC levels [99, 100]. Clearly, further investigations are necessary to determine the underlying mechanisms of elevated MDSC levels in COVID-19 patients.

Importantly, Atanackovic et al. found that severe COVID-19 patients had a larger amount of MDSCs and higher concentrations of TGF-*β*, compared with mild patients. This might result in an undesirable suppression of SARS–CoV-2–specific T cell responses, which can contribute to poor outcomes in these patients (Figure 2(a)) [101]. Therefore, both MDSC and TGF-*β* should be studied further as possible pathogenic/prognostic variables and therapeutic targets in COVID-19 [101]. Moreover, it has been reported that M-MDSC level in the blood was considerably higher in COVID-19 patients, compared with healthy controls [102]. Additionally, it was observed that patients with more severe illnesses had considerably higher peak of M-MDSC frequencies in their blood, compared with mild and healthy controls [102]. Similarly, Kvedaraite et al. found that there were high frequencies of M-MDSC in blood samples from severe COVID-19 patients, compared with moderate patients [97]. Equally important, Jiménez-Cortegana et al. found that the amount of peripheral M-MDSC in COVID-19 patients was significantly increased, compared with healthy controls [8]. They also reported a negative correlation between levels of M-MDSC and activated T cells, implying that M-MDSCs suppress T cell activation [8]. Another study found an association between M-MDSC level and sex and age [103]. Men had significantly higher levels of M-MDSCs, and there was a significant positive correlation between age and M-MDSC level [103]. Moreover, Xue et al. found a strong negative correlation between M-MDSC frequency and lymphocyte levels and serum albumin and a positive correlation with oropharyngeal viral loads and length of hospitalization in severe COVID-19 patients, suggesting that M-MDSC might be used to predict the severity and prognosis of COVID-19 [104]. Additionally, Emsen et al. found that patients with COVID-19 had substantially higher levels of total MDSCs, PMN-MDSCs, and M-MDSCs, when compared to healthy controls [105]. Furthermore, they found that severe COVID-19 patients had much higher PMN-MDSC levels than mild COVID-19 patients [105]. However, another study observed that levels of G-MDSC and M-MDSC were higher in COVID-19 patients, compared with healthy controls, with no difference between COVID-19 severity or ventilator status [106]. Schulte-Schrepping et al. found a large proportion of preneutrophil and immature neutrophil cells in peripheral blood of severe COVID-19 patients, compared with mild patients, demonstrating that a dysregulated myeloid cell component contributes to severe COVID-19 [96]. Moreover, other studies showed that the frequency of PMN-MDSCs in severe COVID-19 patients was higher compared with mild disease or healthy controls [107–109]. Recent studies indicated that PMN-MDSCs played a novel function in platelet activation by decreasing L-arginine concentration in COVID-19 patients, indicating a novel role of MDSCs in the pathogenesis of COVID-19 [110].

#### 3.2.2. Protective Roles

MDSCs have been characterized as a response to inflammatory processes that help to limit overly aggressive and possibly damaging immune responses by inhibiting the function of several immune cells including NK cell and T lymphocytes in severe COVID-19 patients (Figure 2(b)) [111]. It has been reported that the expansion of MDSCs was found to be significantly linked to lymphopenia and increased arginase activity in response to COVID-19 [98]. Surprisingly, *in vitro*, arginine was found to be essential in the lifecycle of several DNA and RNA viruses [112]. Therefore, therapeutic depletion of arginine may inhibit SARS-CoV-2 replication [112]. Accordingly, MDSCs may have a protective role against SARS-CoV-2 by producing ARG1. In addition, Agrati et al. demonstrated that patients with severe COVID-19 had a massive expansion of MDSCs, accounting for up to 90% of the total number of circulating mononuclear cells in the blood, indicating that immunological suppression, potentially mediated by expanded MDSCs, might be useful in decreasing inflammation and lung damage caused by hyperactivated cytotoxic T cells (Figure 2(b)) [113]. Interestingly, Takano *et al*. reported that G-MDSCs, but not other MDSC subgroups, exhibited temporary expansion in severe COVID-19, but not in mild or moderate diseases [114]. This temporary expansion of G-MDSCs was seen among survivors of severe COVID-19, but not among nonsurvivors, suggesting a beneficial effect of the G-MDSCs subgroup, which has the ability to reduce excessive inflammation during severe COVID-19 recovery [114].

In vaccination settings, a study reported that frequency of PMN-MDSCs and M-MDSCs increased dramatically after the first COVID-19 vaccination dose, which was reduced at further periods of time, approaching but not reaching prevaccination values, which may have reduced postvaccination responses [115].

## 4. Targeting Tregs and MDSCs in COVID-19

The primary causes of morbidity and death in COVID-19 patients are cytokine storm and defective haemostasis [2]. Function of Tregs in COVID-19 patients should be evaluated depending on their physiological location and illness stage. If there are more Tregs in the lungs during inflammatory cytokine storm, this could reduce the excessive immune responses [116, 117]. Therefore, expanding Tregs or increasing their activity could be beneficial in this context. There are some potential strategies to expand Tregs or enhance their activity. Tregs cord infusions were associated with recovery in two critical COVID-19 patients; this could be due to increase in Tregs and reduction in hyperinflammation [118]. In type 1 diabetes and autoimmune diseases, low-dose IL-2 has been utilized to induce Treg expansion. There is only one clinical trial investigated the administration of low-dose IL-2 in hospitalized COVID-19 patients with ARDS [119]. This trial has been completed, and results are awaited (trial NCT04357444 registered at ClinicalTrials.gov). Additionally, abatacept (recombinant Fc-fused CTLA-4 protein) could potentially influence innate cell activation, such as monocytes and dendritic cells, and increase Tregs, although research on Treg activity is limited and contradictory [116, 120]. Moreover, abatacept could be an attractive drug to reduce the hyperinflammation condition of severe COVID-19 patients [121].

On the other hand, some studies showed that increased Tregs are associated with inhibiting antiviral T cell responses in the severe stages of COVID-19 [43]. Therefore, reducing Treg levels or suppressing their activity could provide some benefits to COVID-19 patients. In this context, there are some potential strategies to reduce Treg levels or activities. These include the use of monoclonal antibodies targeting Tregs (e.g., anti-CD25 (LMB-2)), immune checkpoint inhibitors (e.g., anti-PD-1, anti-CTLA-4), TGF-*β* blockers to suppress induced Tregs, and denileukin diftitox (DAB-IL-2, ONTAK).

There are some potential strategies to reduce MDSC activity. IL-6 blocker can partially elevate HLA-DR expression, leading to decrease levels of M-MDSC in severe COVID-19 patients [99]. Moreover, expression of vitamin D receptor correlates with the immunosuppressive activity of MDSCs [122]. The active form of vitamin D, 1,25(OH)2D, decreases the suppressive action of MDSCs [122, 123]. Moreover, a clinical trial reported that in hospitalized COVID-19 patients with acute respiratory distress syndrome, administration of L-citrulline, an endogenous precursor of arginine, for one week, decreases the possibility of organ failure, compared to the placebo group (trial NCT04404426 registered at ClinicalTrials.gov).

## 5. Perspective

It is evident that Tregs are different in patients with different disease severities. Treg increase in the early stages of COVID-19 could be one of the viral evasion mechanisms to inhibit antiviral immune responses. Therefore, approaches to target Tregs and reducing their suppressive activity could be useful to restore antiviral immune responses, especially in old patients with immune-compromised immunity. When the disease progresses, Tregs are beneficial to inhibit inflammation; however, Tregs are either reduced or nonfunctional in the periphery or lung of severe COVID-19 patients; unfortunately, this is part of the hard battle between the virus and the immune system. It is important to exploit approaches inducing or expanding Tregs in these patients to reduce hyperinflammation and tissue damage.

Mechanisms of Treg reduction in peripheral blood of severe COVID-19 patients are largely unknown, but migration of Tregs to the lung to inhibit inflammation and protect tissue damage could be a potential mechanism. Another mechanism could be Treg apoptosis because of deficiency in Treg growth cytokines such as IL-2. There are no enough strong studies with large number of patients reported on the level and function of Tregs in the lung. One study found that Tregs in the lung of severe COVID-19 patients downregulated FoxP3 expression, and they were of a more activated rather than a suppressive phenotype, which could induce hyperinflammation and tissue damage [63].

Most available studies reported elevated levels of MDSCs in severe COVID-19 patients. Identification of MDSC's subpopulations in severe versus mild or asymptomatic COVID-19 patients is essential for prognosis and therapeutic targeting. MDSCs, by releasing ARG1, are a double-edged sword, which inhibit T cell proliferation through decreasing L-arginine, but also reduce SARS-CoV-2 replication. Further studies are needed to investigate MDSC subpopulations and the effect of targeting them in severe COVID-19 patients.

## 6. Conclusions

Studies investigating Tregs in COVID-19 patients have reported different results. Some studies reported that Tregs were increased in COVID-19 patients and played a negative role in the progression of the disease. Other studies reported that the decreased levels of Tregs and increased response of proinflammatory cytokines in COVID-19 could be associated with hyperinflammation and severe disease. However, an early increase in inflammatory cytokines could be associated with faster viral clearance and early immune responses. The balance between anti-inflammatory cells such as Tregs and proinflammatory cells such as Th17 is critical to determine the clinical outcome in COVID-19 patients. Understanding the relationship between Tregs and inflammatory cytokines could lead to discovering novel therapeutic approaches in COVID-19 disease.

Different studies observed that alteration in MDSC levels in the blood has been associated with disease severity. Furthermore, there is a strong negative correlation between M-MDSC frequency and lymphocyte levels in severe COVID-19 patients, suggesting that M-MDSC might be used to predict the severity and prognosis of COVID-19. Moreover, different studies observed that the frequency of G-MDSCs was higher in severe COVID-19 and might be associated with lymphopenia and severity of the disease. However, some studies found that MDSCs were expanded as a response to inflammatory processes, which helped to limit an overly aggressive and possibly damaging immune responses by inhibiting several immune cells. In addition, some researchers found that G-MDSCs, but not other MDSC subgroups, exhibited temporary expansion in severe COVID-19, which can reduce excessive inflammation during severe COVID-19.

In conclusion, immune cells, including Tregs and MDSCs, should be further studied as potential prognostic biomarkers and therapeutic targets in COVID-19. Further studies, including higher numbers of patients with mild and severe diseases and validated protocols for identification and measuring levels of Tregs and MDSCs, are required to make stronger conclusions. Additionally, investigations on patients receiving different types of COVID-19 vaccinations are urgently needed to determine any changes in Treg and MDSC levels in circulation following vaccinations, and how these changes can correlate with the protective roles of vaccines.

## Figures and Tables

**Table 1:** Summary of Tregs in COVID-19 patients with different severities.

	Study groups (number of patients)	Change in Treg levels	Cell phenotype	Notes	Reference no.
Xie et al.	Asymptomatic disease	Decrease	NA	In asymptomatic patients, IL-2 was associated with faster viral clearance and early immune responses.	[54]
Kratzer et al.	Convalescent patients (109) vs. healthy (98)	Decrease	CD25^+^FoxP3^+^	Acute SARS-CoV-2 infection is beneficial by activation of T cells or harmful by reduction of neutrophils.	[51]
Chen et al.	Mild (80)/severe (22) vs. healthy (67)	Increase	CD4^+^CD25^+^CD127^low^	CD4^+^ T cells, B cells, IL-6, and IL-10 are indicators of COVID-19 severity.	[47]
Sadeghi et al.	Critical (40) vs. healthy (40)	Decrease	CD4^+^CD25^+^CD127^−^	Imbalanced ratios of Th17/Tregs could play an important role in inflammatory responses and the pathogenesis of the disease.	[52]
Jiménez-Cortegana et al.	Hospitalized (20) vs. healthy (20)	Decrease	CD4^+^CD25^high^CD127^−^	M-MDSCs, but not Tregs, could play a role in the immunosuppression shown in COVID-19 patients.	[8]
Patterson et al.	Different severity (224)	Decrease	NA	Decreased Tregs in COVID-19 compared with healthy controls.	[53]
Mohebbi et al.	Different severity (30)	Decrease	CD4^+^FoxP3^+^CD25^+^	Decreased Tregs in COVID-19 patients compared with healthy controls.	[57]
Galván-Peña et al.	Different severity (57)	Increase	CD25^+^FoxP3^+^	Increased Tregs in severe patients is associated with worse outcome.	[43]
Kalfaoglu et al.	Severe	Decrease	NA	In the lung, T cells highly expressed immune-regulatory receptors and CD25, while suppressing expression of FoxP3.	[63]
Qin et al.	Severe (286) vs. non-severe (166)	Decrease	CD4^+^CD25^+^CD127^low^		[59]
Neumann et al.	Severe (20) vs. mild/moderate (23)	Increase	IL-10-secreting Tregs		[45]
Wang et al.	Extremely severe (15) vs. severe (20) vs. mild (30)	Decrease	CD45RA^+^ cells	The percentage of natural Tregs was decreased in extremely severe patients.	[61]
Wang et al.	Critical (3) vs. severe (5) vs. mild (4)	Decrease	CD4^+^CD25^+^CD127^−^	Tregs increase during progression from mild to severe then decreased through the progression to critical disease.	[62]
Meckiff et al.	Hospitalized (critical) vs. non hospitalized (mild)	Decrease	NA		[55]
Chen et al.	Severe (11) vs. moderate (10)	Decrease	CD4^+^CD25^+^CD127^low^ and CD45RA^+^		[60]
Rezaei et al.	Critical (8) vs. severe (27) vs. Moderate (17)	No change	CD4^+^CD25^+^FOXP3^+^		[50]
Rutkowska et al.	Critical (18) vs. severe (23)	Decrease	NA	Percentage of CD45RO^+^CD95^+^ Tregs, among other Treg subsets, was higher in critical compared to severe.	[56]
Ronit et al.	Mechanically ventilated patients (4) with moderate-to-severe COVID-19 ARDS	Increase	FoxP3^+^CTLA-4^+^ Tregs	Increased Tregs with activation markers in the lung.	[46]
Vicket et al.	SARS-CoV-2 (24) vs. RSV (10) vs. Flu (9) vs. Healthy donors (23)	Immune landscape in SARS cov-2 similar to flu or RSV patients	CD25^+^ CD127^−^Foxp3^+^	Only in critical patients, the levels of CD25^+^CD127^−^ FoxP3^+^ cells were increased.	[44]

NA: not available.

## Abbreviations

ARG: Arginase
ARDS: Acute respiratory distress syndrome
COVID-19: Coronavirus disease 2019
CTLA-4: Cytotoxic T lymphocyte associated antigen-4
E-MDSCs: Early stage myeloid- derived suppressor cells
FoxP3: Forkhead box P3
G-MDSCs: Granulocytic myeloid- derived suppressor cells
HCV: Hepatitis C virus
HBV: Hepatitis B virus
HIV: Human immunodeficiency virus
HSV-2: Herpes simplex virus type 2
IL: Interleukin
MDSCs: Myeloid-derived suppressor cells
M-MDSCs: Monocytic myeloid- derived suppressor cells
NOS: Nitric oxide synthase
NK: Natural killer
ONOO^−^: Peroxynitrite
PD-1: Programmed cell death protein 1
PMN-MDSCs: Polymorphonuclear myeloid-derived suppressor cells
RSV: Respiratory syncytial virus
SARS-CoV-2: Severe acute respiratory syndrome coronavirus 2
TGF-*β*: Transforming growth factor –Beta 1
Th2: T helper 2
Th17: T helper 17
tTreg: Thymus-derived Tregs
Tregs: T regulatory cells
pTreg: Peripherally-T regulatory cells
WBCs: White blood cells.

## References

[1] Chan J. F.-W., Yuan S., Kok K. H. (2020). A familial cluster of pneumonia associated with the 2019 novel coronavirus indicating person-to-person transmission: a study of a family cluster. *The Lancet*.

[2] Huang C., Wang Y., Li X. (2020). Clinical features of patients infected with 2019 novel coronavirus in Wuhan, China. *The Lancet*.

[3] Gandhi R. T., Lynch J. B., Del Rio C. (2020). Mild or moderate Covid-19. *New England Journal of Medicine*.

[4] Berlin D. A., Gulick R. M., Martinez F. J. (2020). Severe COVID-19. *New England Journal of Medicine*.

[5] Thakur B., Dubey P., Benitez J. (2021). A systematic review and meta-analysis of geographic differences in comorbidities and associated severity and mortality among individuals with COVID-19. *Scientific Reports*.

[6] Liao D., Zhou F., Luo L. (2020). Haematological characteristics and risk factors in the classification and prognosis evaluation of COVID-19: a retrospective cohort study. *The Lancet Haematology*.

[7] Siddiqi H. K., Mehra M. R. (2020). COVID-19 illness in native and immunosuppressed states: a clinical-therapeutic staging proposal. *The Journal of Heart and Lung Transplantation*.

[8] Jiménez-Cortegana C., Liró J., Palazón-Carrión N. (2021). Increased blood monocytic myeloid derived suppressor cells but low regulatory T lymphocytes in patients with mild COVID-19. *Viral Immunology*.

[9] O'Connor M. A., Rastad J. L., Green W. R. (2017). The role of myeloid-derived suppressor cells in viral infection. *Viral Immunology*.

[10] Bowers N. L., Helton E. S., Huijbregts R. P. H., Goepfert P. A., Heath S. L., Hel Z. (2014). Immune suppression by neutrophils in HIV-1 infection: role of PD-L1/PD-1 pathway. *PLoS Pathogens*.

[11] Thakuri B. K. C., Zhang J., Zhao J. (2020). LncRNA HOTAIRM1 promotes MDSC expansion and suppressive functions through the HOXA1-miR124 axis during HCV infection. *Scientific Reports*.

[12] Sakaguchi S., Miyara M., Costantino C. M., Hafler D. A. (2010). FOXP3^+^ regulatory T cells in the human immune system. *Nature Reviews Immunology*.

[13] Tang Q., Bluestone J. A. (2013). Regulatory T-cell therapy in transplantation: moving to the clinic. *Cold Spring Harbor Perspectives in Medicine*.

[14] Xu A., Liu Y., Chen W. (2016). TGF-*β*–induced regulatory T cells directly suppress B cell responses through a noncytotoxic mechanism. *The Journal of Immunology*.

[15] Zheng S. G., Wang J. H., Gray J. D., Soucier H., Horwitz D. A. (2004). Natural and induced CD4+ CD25+ cells educate CD4+ CD25− cells to develop suppressive activity: the role of IL-2, TGF-*β*, and IL-10. *The Journal of Immunology*.

[16] Hsieh C.-S., Liang Y., Tyznik A. J., Self S. G., Liggitt D., Rudensky A. Y. (2004). Recognition of the peripheral self by naturally arising CD25^+^ CD4^+^ T cell receptors. *Immunity*.

[17] Tsuji N. M., Mizumachi K., Kurisaki J. I. (2003). Antigen-specific, CD4+ CD25+ regulatory T cell clones induced in Peyer’s patches. *International Immunology*.

[18] Abbas A. K., Benoist C., Bluestone J. A. (2013). Regulatory T cells: recommendations to simplify the nomenclature. *Nature Immunology*.

[19] Josefowicz S. Z., Lu L.-F., Rudensky A. Y. (2012). Regulatory T cells: mechanisms of differentiation and function. *Annual Review of Immunology*.

[20] Bluestone J. A., Abbas A. K. (2003). Natural versus adaptive regulatory T cells. *Nature Reviews Immunology*.

[21] Wan Y. Y., Flavell R. A. (2007). Regulatory T-cell functions are subverted and converted owing to attenuated Foxp3 expression. *Nature*.

[22] Miyara M., Yoshioka Y., Kitoh A. (2009). Functional delineation and differentiation dynamics of human CD4^+^ T cells expressing the FoxP3 transcription factor. *Immunity*.

[23] Zhao J., Zhao J., Perlman S. (2012). Differential effects of IL-12 on Tregs and non-Treg T cells: roles of IFN-*γ*, IL-2 and IL-2R. *PLoS One*.

[24] Dittmer U., He H., Messer R. J. (2004). Functional impairment of CD8^+^ T cells by regulatory T cells during persistent retroviral infection. *Immunity*.

[25] Suvas S., Kumaraguru U., Pack C. D., Lee S., Rouse B. T. (2003). CD4+ CD25+ T cells regulate virus-specific primary and memory CD8+ T cell responses. *The Journal of Experimental Medicine*.

[26] Veiga-Parga T., Sehrawat S., Rouse B. T. (2013). Role of regulatory T cells during virus infection. *Immunological Reviews*.

[27] Raiden S., Pandolfi J., Payasliàn F. (2014). Depletion of circulating regulatory T cells during severe respiratory syncytial virus infection in young children. *American Journal of Respiratory and Critical Care Medicine*.

[28] Li B., Wu F. L., Feng X. B., Sun D. K., Cui Q. Q., Zhao Z. X. (2012). Changes and the clinical significance of CD4+ CD25+ regulatory T cells and Th17 cells in peripheral blood of infants with respiratory syncytial virus bronchiolitis. *Chinese Journal of Cellular and Molecular Immunology*.

[29] Qin L., Hu C. P., Feng J. T., Xia Q. (2011). Activation of lymphocytes induced by bronchial epithelial cells with prolonged RSV infection. *PLoS One*.

[30] Sugimoto K., Ikeda F., Stadanlick J., Nunes F. A., Alter H. J., Chang K. M. (2003). Suppression of HCV-specific T cells without differential hierarchy demonstrated _ex vivo_ in persistent HCV infection. *Hepatology*.

[31] Cabrera R., Tu Z., Xu Y. (2004). An immunomodulatory role for CD4+ CD25+ regulatory T lymphocytes in hepatitis C virus infection. *Hepatology*.

[32] Boettler T., Spangenberg H. C., Neumann-Haefelin C. (2005). T cells with a CD4+ CD25+ regulatory phenotype suppress in vitro proliferation of virus-specific CD8+ T cells during chronic hepatitis C virus infection. *Journal of Virology*.

[33] Claassen M. A., de Knegt R. J., Tilanus H. W., Janssen H. L. A., Boonstra A. (2010). Abundant numbers of regulatory T cells localize to the liver of chronic hepatitis C infected patients and limit the extent of fibrosis. *Journal of Hepatology*.

[34] Wan Z., Zhou Z., Liu Y. (2020). Regulatory T cells and T helper 17 cells in viral infection. *Scandinavian Journal of Immunology*.

[35] Wu W., Li J., Chen F., Zhu H., Peng G., Chen Z. (2010). Circulating Th17 cells frequency is associated with the disease progression in HBV infected patients. *Journal of Gastroenterology and Hepatology*.

[36] Feng H., Yin J., Han Y. P. (2015). Regulatory T cells and IL-17+ T helper cells enhanced in patients with chronic hepatitis B virus infection. *International Journal of Clinical and Experimental Medicine*.

[37] Fu J., Xu D. P., Zhao P. (2006). The characterization of regulatory T cells in peripheral blood of HBV-infected patients. *Zhonghua Yi Xue Za Zhi*.

[38] Yang G., Liu A., Xie Q. (2007). Association of CD4+ CD25+ Foxp3+ regulatory T cells with chronic activity and viral clearance in patients with hepatitis B. *International Immunology*.

[39] Stoop J. N., van der Molen R. G., Baan C. C. (2005). Regulatory T cells contribute to the impaired immune response in patients with chronic hepatitis B virus infection. *Hepatology*.

[40] Prendergast A., Prado J. G., Kang Y. H. (2010). HIV-1 infection is characterized by profound depletion of CD161+ Th17 cells and gradual decline in regulatory T cells. *AIDS*.

[41] Wang W. H., Ming L., Wang Y., Kan Q. C., Zhang X. Y. (2013). High frequency of regulatory T cells among HIV type 1-infected men who have sex with men correlates with disease progression. *Chinese Medical Journal*.

[42] Milman N., Zhu J., Johnston C. (2016). In situ detection of regulatory T cells in human genital herpes simplex virus type 2 (HSV-2) reactivation and their influence on spontaneous HSV-2 reactivation. *The Journal of Infectious Diseases*.

[43] Galván-Peña S., Leon J., Chowdhary K. (2021). Profound Treg perturbations correlate with COVID-19 severity. *Proceedings of the National Academy of Sciences*.

[44] Vick S. C., Frutoso M., Mair F. (2021). A regulatory T cell signature distinguishes the immune landscape of COVID-19 patients from those with other respiratory infections. *Science Advances*.

[45] Neumann J., Prezzemolo T., Vanderbeke L. (2020). Increased IL-10-producing regulatory T cells are characteristic of severe cases of COVID-19. *Clinical & translational immunology*.

[46] Ronit A., Berg R. M. G., Bay J. T. (2021). Compartmental immunophenotyping in COVID-19 ARDS: a case series. *Journal of Allergy and Clinical Immunology*.

[47] Chen X., Huang J., Huang Y. (2020). Characteristics of immune cells and cytokines in patients with coronavirus disease 2019 in Guangzhou, China. *Human Immunology*.

[48] Yang J., Zhang E., Zhong M. (2020). Impaired T cell functions along with elevated activated Tregs at the early stage of asymptomatic SARS-CoV-2 infection. *SSRN Electronic Journal*.

[49] De Biasi S., Meschiari M., Gibellini L. (2020). Marked T cell activation, senescence, exhaustion and skewing towards TH17 in patients with COVID-19 pneumonia. *Nature Communications*.

[50] Rezaei M., Marjani M., Mahmoudi S., Mortaz E., Mansouri D. (2021). Dynamic changes of lymphocyte subsets in the course of covid-19. *International Archives of Allergy and Immunology*.

[51] Kratzer B., Trapin D., Ettel P. (2021). Immunological imprint of COVID-19 on human peripheral blood leukocyte populations. *Allergy*.

[52] Sadeghi A., Tahmasebi S., Mahmood A. (2021). Th17 and Treg cells function in SARS-CoV2 patients compared with healthy controls. *Journal of Cellular Physiology*.

[53] Patterson B. K., Guevara-Coto J., Yogendra R. (2021). Immune-based prediction of COVID-19 severity and chronicity decoded using machine learning. *Frontiers in Immunology*.

[54] Xie C., Li Q., Li L. (2021). Association of early inflammation with age and asymptomatic disease in COVID-19. *Journal of Inflammation Research*.

[55] Meckiff B. J., Ramírez-Suástegui C., Fajardo V. (2020). Imbalance of regulatory and cytotoxic SARS-CoV-2-reactive CD4^+^ T cells in COVID-19. *Cell*.

[56] Rutkowska E., Kwiecień I., Żabicka M. (2021). Cytokines and leukocytes subpopulations profile in SARS-CoV-2 patients depending on the CT score severity. *Viruses*.

[57] Mohebbi S. R., Baghaei K., Rostami-Nejad M. (2020). Significant changes of CD4, FOXP3, CD25, and IL6 expression level in Iranian COVID-19 patients. *Gastroenterology and Hepatology From Bed to Bench*.

[58] Jia R., Wang X., Liu P. (2020). Mild cytokine elevation, moderate CD4+ T cell response and abundant antibody production in children with COVID-19. *Virologica Sinica*.

[59] Qin C., Zhou L., Hu Z. (2020). Dysregulation of immune response in patients with coronavirus 2019 (COVID-19) in Wuhan, China. *Clinical Infectious Diseases*.

[60] Chen G., Wu D., Guo W. (2020). Clinical and immunological features of severe and moderate coronavirus disease 2019. *The Journal of Clinical Investigation*.

[61] Wang F., Hou H., Luo Y. (2020). The laboratory tests and host immunity of COVID-19 patients with different severity of illness. *JCI Insight*.

[62] Wang W., Su B., Pang L. (2020). High-dimensional immune profiling by mass cytometry revealed immunosuppression and dysfunction of immunity in COVID-19 patients. *Cellular & Molecular Immunology*.

[63] Kalfaoglu B., Almeida-Santos J., Tye C. A., Satou Y., Ono M. (2020). T-cell hyperactivation and paralysis in severe COVID-19 infection revealed by single-cell analysis. *Frontiers in Immunology*.

[64] Yang X., Yu Y., Xu J. (2020). Clinical course and outcomes of critically ill patients with SARS-CoV-2 pneumonia in Wuhan, China: a single-centered, retrospective, observational study. *The Lancet Respiratory Medicine*.

[65] Wang W., Thomas R., Oh J., Su D. M. (2021). Thymic aging may be associated with COVID-19 pathophysiology in the elderly. *Cell*.

[66] Oh J., Wang W., Thomas R., Su D. M. (2017). Capacity of tTreg generation is not impaired in the atrophied thymus. *PLoS Biology*.

[67] Raynor J., Lages C. S., Shehata H., Hildeman D. A., Chougnet C. A. (2012). Homeostasis and function of regulatory T cells in aging. *Current Opinion in Immunology*.

[68] Tsukamoto H., Clise-Dwyer K., Huston G. E. (2009). Age-associated increase in lifespan of naive CD4 T cells contributes to T-cell homeostasis but facilitates development of functional defects. *Proceedings of the National Academy of Sciences*.

[69] Chougnet C. A., Tripathi P., Lages C. S. (2011). A major role for Bim in regulatory T cell homeostasis. *The Journal of Immunology*.

[70] Sacchi A., Grassi G., Bordoni V. (2020). Early expansion of myeloid-derived suppressor cells inhibits SARS-CoV-2 specific T-cell response and may predict fatal COVID-19 outcome. *Cell Death & Disease*.

[71] Talmadge J. E., Gabrilovich D. I. (2013). History of myeloid-derived suppressor cells. *Nature Reviews. Cancer*.

[72] Gabrilovich D. I. (2017). Myeloid-derived suppressor cells. *Cancer Immunology Research*.

[73] Rajabinejad M., Salari F., Gorgin Karaji A., Rezaiemanesh A. (2019). The role of myeloid-derived suppressor cells in the pathogenesis of rheumatoid arthritis; anti- or pro-inflammatory cells?. *Artificial Cells, Nanomedicine, and Biotechnology*.

[74] Kumar V., Patel S., Tcyganov E., Gabrilovich D. I. (2016). The nature of myeloid-derived suppressor cells in the tumor microenvironment. *Trends in Immunology*.

[75] Toor S. M., Khalaf S., Murshed K., Abu Nada M., Elkord E. (2020). Myeloid cells in circulation and tumor microenvironment of colorectal cancer patients with early and advanced disease stages. *Journal of Immunology Research*.

[76] Gabrilovich D. I., Nagaraj S. (2009). Myeloid-derived suppressor cells as regulators of the immune system. *Nature Reviews Immunology*.

[77] Koushki K., Salemi M., Miri S. M., Arjeini Y., Keshavarz M., Ghaemi A. (2021). Role of myeloid-derived suppressor cells in viral respiratory infections; hints for discovering therapeutic targets for COVID-19. *Biomedicine & Pharmacotherapy*.

[78] Condamine T., Gabrilovich D. I. (2011). Molecular mechanisms regulating myeloid-derived suppressor cell differentiation and function. *Trends in Immunology*.

[79] Huang B., Pan P. Y., Li Q. (2006). Gr-1+CD115+ immature myeloid suppressor cells mediate the development of tumor-induced T regulatory cells and T-cell anergy in tumor-bearing host. *Cancer Research*.

[80] Cassetta L., Bruderek K., Skrzeczynska-Moncznik J. (2020). Differential expansion of circulating human MDSC subsets in patients with cancer, infection and inflammation. *Journal for Immunotherapy of Cancer*.

[81] Wang L., Chang E. W. Y., Wong S. C., Ong S. M., Chong D. Q. Y., Ling K. L. (2013). Increased myeloid-derived suppressor cells in gastric cancer correlate with cancer stage and plasma S100A8/A9 proinflammatory proteins. *Journal of Immunology*.

[82] Mandruzzato S., Solito S., Falisi E. (2009). IL4R*α*+myeloid-derived suppressor cell expansion in cancer patients. *Journal of Immunology*.

[83] Liu C. Y., Wang Y. M., Wang C. L. (2010). Population alterations of L-arginase- and inducible nitric oxide synthase-expressed CD11b+/CD14^−^/CD15+/CD33+ myeloid-derived suppressor cells and CD8+ T lymphocytes in patients with advanced-stage non-small cell lung cancer. *Journal of Cancer Research and Clinical Oncology*.

[84] Vuk-Pavlović S., Bulur P. A., Lin Y. (2010). Immunosuppressive CD14+HLA-DRlow/− monocytes in prostate cancer. *Prostate*.

[85] Khaled Y. S., Ammori B. J., Elkord E. (2014). Increased levels of granulocytic myeloid-derived suppressor cells in peripheral blood and tumour tissue of pancreatic cancer patients. *Journal of Immunology Research*.

[86] Lindau D., Gielen P., Kroesen M., Wesseling P., Adema G. J. (2013). The immunosuppressive tumour network: myeloid-derived suppressor cells, regulatory T cells and natural killer T cells. *Immunology*.

[87] Motallebnezhad M., Jadidi-Niaragh F., Qamsari E. S., Bagheri S., Gharibi T., Yousefi M. (2016). The immunobiology of myeloid-derived suppressor cells in cancer. *Tumour Biology*.

[88] Cai W., Qin A., Guo P. (2013). Clinical significance and functional studies of myeloid-derived suppressor cells in chronic hepatitis C patients. *Journal of Clinical Immunology*.

[89] Norris B. A., Uebelhoer L. S., Nakaya H. I., Price A. A., Grakoui A., Pulendran B. (2013). Chronic but not acute virus infection induces sustained expansion of myeloid suppressor cell numbers that inhibit viral-specific T cell immunity. *Immunity*.

[90] Tacke R. S., Lee H. C., Goh C. (2012). Myeloid suppressor cells induced by hepatitis C virus suppress T-cell responses through the production of reactive oxygen species. *Hepatology*.

[91] Ren J. P., Zhao J., Dai J. (2016). Hepatitis C virus-induced myeloid-derived suppressor cells regulate T-cell differentiation and function via the signal transducer and activator of transcription 3 pathway. *Immunology*.

[92] Garg A., Spector S. A. (2014). HIV type 1 gp120-induced expansion of myeloid derived suppressor cells is dependent on interleukin 6 and suppresses immunity. *The Journal of Infectious Diseases*.

[93] Vollbrecht T., Stirner R., Tufman A. (2012). Chronic progressive HIV-1 infection is associated with elevated levels of myeloid-derived suppressor cells. *AIDS*.

[94] Pal S., Nandi M., Dey D. (2019). Myeloid-derived suppressor cells induce regulatory T cells in chronically HBV infected patients with high levels of hepatitis B surface antigen and persist after antiviral therapy. *Alimentary Pharmacology & Therapeutics*.

[95] Pallett L. J., Gill U. S., Quaglia A. (2015). Metabolic regulation of hepatitis B immunopathology by myeloid-derived suppressor cells. *Nature Medicine*.

[96] Schulte-Schrepping J., Reusch N., Paclik D. (2020). *Suppressive myeloid cells are a hallmark of severe COVID-19*.

[97] Kvedaraite E., Hertwig L., Sinha I. (2020). *Perturbations in the mononuclear phagocyte landscape associated with COVID-19 disease severity*.

[98] Reizine F., Lesouhaitier M., Gregoire M. (2021). SARS-CoV-2-induced ARDS associates with MDSC expansion, lymphocyte dysfunction, and arginine shortage. *Journal of Clinical Immunology*.

[99] Giamarellos-Bourboulis E. J., Netea M. G., Rovina N. (2020). Complex immune dysregulation in COVID-19 patients with severe respiratory failure. *Cell Host & Microbe*.

[100] Veglia F., Sanseviero E., Gabrilovich D. I. (2021). Myeloid-derived suppressor cells in the era of increasing myeloid cell diversity. *Nature Reviews. Immunology*.

[101] Atanackovic D., Avila S. V., Lutfi F. (2021). Deep dissection of the antiviral immune profile of patients with COVID-19. *Communications Biology*.

[102] Falck-Jones S., Vangeti S., Yu M. (2020). *Functional myeloid-derived suppressor cells expand in blood but not airways of COVID-19 patients and predict disease severity*.

[103] Falck-Jones S., Vangeti S., Yu M. (2021). Functional monocytic myeloid-derived suppressor cells increase in blood but not airways and predict COVID-19 severity. *The Journal of Clinical Investigation*.

[104] Xue G., Jiang M., Zhao R., le A. P., Li J. M. (2021). Elevated frequencies of CD14+HLA-DRlo/neg MDSCs in COVID-19 patients. *Aging (Albany NY)*.

[105] Emsen A., Sumer S., Tulek B. (2022). Correlation of myeloid-derived suppressor cells with C-reactive protein, ferritin and lactate dehydrogenase levels in patients with severe COVID-19. *Scandinavian Journal of Immunology*.

[106] Mortaz E., Dezfuli N. K., Roofchayee N. D., Adcock I. (2021). Myeloid-derived suppressor cells in the blood of COVID-19 patients. *European Respiratory Journal*.

[107] Peñaloza H. F., Lee J. S., Ray P. (2021). Neutrophils and lymphopenia, an unknown axis in severe COVID-19 disease. *PLoS Pathogens*.

[108] Cabrera L. E., Pekkarinen P. T., Alander M. (2021). Characterization of low-density granulocytes in COVID-19. *PLoS Pathogens*.

[109] Thompson E. A., Cascino K., Ordonez A. A. (2021). Metabolic programs define dysfunctional immune responses in severe COVID-19 patients. *Cell Reports*.

[110] Sacchi A., Grassi G., Notari S. (2021). Expansion of myeloid derived suppressor cells contributes to platelet activation by L-arginine deprivation during SARS-CoV-2 infection. *Cell*.

[111] Bordoni V., Sacchi A., Cimini E. (2020). An inflammatory profile correlates with decreased frequency of cytotoxic cells in coronavirus disease 2019. *Clinical Infectious Diseases*.

[112] Grimes J. M., Khan S., Badeaux M., Rao R. M., Rowlinson S. W., Carvajal R. D. (2021). Arginine depletion as a therapeutic approach for patients with COVID-19. *International Journal of Infectious Diseases*.

[113] Agrati C., Sacchi A., Bordoni V. (2020). Expansion of myeloid-derived suppressor cells in patients with severe coronavirus disease (COVID-19). *Cell Death and Differentiation*.

[114] Takano T., Matsumura T., Adachi Y. (2021). Myeloid cell dynamics correlating with clinical outcomes of severe COVID-19 in Japan. *International Immunology*.

[115] Sindhi R., Ashokkumar C., Singh V. (2021). *Cellular and Antibody Immunity after COVID-19 Vaccination at >4-Month Follow Up in Immunocompetent and Immunocompromised Subjects*.

[116] Langdon K., Haleagrahara N. (2018). Regulatory T-cell dynamics with abatacept treatment in rheumatoid arthritis. *International Reviews of Immunology*.

[117] Michelena X., Borrell H., López-Corbeto M. (2020). Incidence of COVID-19 in a cohort of adult and paediatric patients with rheumatic diseases treated with targeted biologic and synthetic disease-modifying anti-rheumatic drugs. *Seminars in Arthritis and Rheumatism*.

[118] Gladstone D. E., Kim B. S., Mooney K., Karaba A. H., D'Alessio F. R. (2020). Regulatory T cells for treating patients with COVID-19 and acute respiratory distress syndrome: two case reports. *Annals of Internal Medicine*.

[119] Hartemann A., Bensimon G., Payan C. A. (2013). Low-dose interleukin 2 in patients with type 1 diabetes: a phase 1/2 randomised, double-blind, placebo-controlled trial. *The Lancet Diabetes & Endocrinology*.

[120] Bonelli M., Göschl L., Blüml S. (2016). Abatacept (CTLA-4Ig) treatment reduces T cell apoptosis and regulatory T cell suppression in patients with rheumatoid arthritis. *Rheumatology*.

[121] Stephen-Victor E., Das M., Karnam A., Pitard B., Gautier J. F., Bayry J. (2020). Potential of regulatory T-cell-based therapies in the management of severe COVID-19. *European Respiratory Journal*.

[122] Kloc M., Ghobrial R. M., Lipińska-Opałka A. (2021). Effects of vitamin D on macrophages and myeloid-derived suppressor cells (MDSCs) hyperinflammatory response in the lungs of COVID-19 patients. *Cellular Immunology*.

[123] Fleet J. C., Burcham G. N., Calvert R. D., Elzey B. D., Ratliff T. L. (2020). 1*α*, 25 Dihydroxyvitamin D (1,25(OH)_2_D) inhibits the T cell suppressive function of myeloid derived suppressor cells (MDSC). *The Journal of Steroid Biochemistry and Molecular Biology*.

